# Prognostic utility of circulating tumor DNA assessment in immune checkpoint inhibitor-treated advanced non-small cell lung cancer: a systematic review and meta-analysis

**DOI:** 10.3389/fimmu.2026.1812209

**Published:** 2026-06-09

**Authors:** Yulin Wang, Shuang Wu, Yanfang Wei, Shize Fan, Zihui Xu, Dongyang Li, Zan Teng, Jin Wang

**Affiliations:** 1 Department of Medical Oncology, the First Hospital of China Medical University, Shenyang, China; 2 Provincial Key Laboratory of Anticancer Drugs and Biotherapy of Liaoning Province, the First Hospital of China Medical University, Shenyang, China; 3 Clinical Cancer Research Center of Shenyang, the First Hospital of China Medical University, Shenyang, China; 4 Department of Endocrinology, the First Hospital of China Medical University, Shenyang, China

**Keywords:** circulating tumor DNA, immune checkpoint inhibitors, immunotherapy, meta-analysis, non-small cell lung cancer

## Abstract

**Objective:**

This meta-analysis aimed to assess the prognostic value of circulating tumor DNA (ctDNA) in predicting progression-free survival (PFS) and overall survival (OS) of advanced non-small cell lung cancer (NSCLC) patients treated with immune checkpoint inhibitors (ICIs), thereby providing evidence-based support for clinical decision-making.

**Methods:**

Six major English and Chinese databases (PubMed, Embase, Cochrane Library, etc.) were scanned from inception to August 2025. Studies evaluating the association between ctDNA and survival outcomes in advanced NSCLC patients receiving ICIs were included. Thirty-one eligible studies (2,107 patients) were selected following predefined criteria. Study quality was evaluated using Cochrane RoB-2 and the Newcastle-Ottawa Scale (NOS). Hazard ratios (HRs) and 95% confidence intervals (95% CIs) were pooled using random/fixed-effects models. Heterogeneity was assessed via Cochran’s Q test and I^2^ statistics. Subgroup analyses, sensitivity tests, and funnel plots were conducted to evaluate robustness and publication bias.

**Results:**

Patients with undetectable ctDNA at baseline showed significantly prolonged PFS (HR = 0.49, 95% CI: 0.34–0.70, P < 0.01) and OS (HR = 0.45, 95% CI: 0.32–0.65, P < 0.01). Patients achieving ctDNA reduction or response during treatment exhibited substantial PFS (HR = 0.27, 95% CI: 0.21–0.35, P < 0.01) and OS (HR = 0.23, 95% CI: 0.17–0.31, P < 0.01) benefits, with complete molecular response (100% reduction in variant allele frequency, VAF) demonstrating the strongest predictive power, characterized by the lowest heterogeneity and favorable HRs for PFS (HR = 0.27, 95% CI: 0.18–0.41, P < 0.01) and OS (HR = 0.19, 95% CI: 0.12–0.29, P < 0.01). Subgroup analyses revealed three key patterns: superior predictive value in combination therapy versus monotherapy; higher sensitivity of next-generation sequencing (NGS) over digital polymerase chain reaction (PCR); and enhanced predictive power in later-line therapy compared to first-line therapy. Sensitivity analyses confirmed the stability of the results.

**Conclusion:**

Baseline ctDNA status and early dynamic changes are reliable prognostic indicators in advanced NSCLC patients receiving ICI therapy, particularly in combination regimens and cases achieving complete molecular clearance. These findings support ctDNA as a biomarker for personalized treatment strategies.

**Clinical Trial Registration:**

https://www.crd.york.ac.uk/PROSPERO, CRD420251025308.

## 1 Introduction

Lung cancer represents the second most prevalent malignancy globally and is the leading cause of cancer-related mortality (1). Non-small cell lung cancer (NSCLC) constitutes approximately 85% of all lung cancer cases, with patients facing a poor prognosis; over 50% are diagnosed at an advanced stage (2, 3). The introduction of immune checkpoint inhibitors (ICIs) has transformed the therapeutic landscape for NSCLC. Compared to chemotherapy or radiotherapy, ICIs significantly prolong survival in advanced NSCLC patients, demonstrating durable clinical responses and a favorable safety profile (4, 5). However, the objective response to immunotherapy is observed only in a subset of patients, driving ongoing breakthroughs in biomarker research to optimize patient stratification and treatment efficacy.

Current detection of three U.S. Food and Drug Administration-approved predictive biomarkers for immunotherapy — programmed cell death ligand-1 (PD-L1) (6), microsatellite instability (MSI) (7, 8), and tissue tumor mutational burden (7) — all require tissue samples. However, these tests are limited by tumor heterogeneity and suboptimal sensitivity, hindering precise identification of early-stage tumors and dynamic monitoring of tumor evolution, and showing inadequate efficacy in detecting minimal residual disease.

Liquid biopsy technology overcomes the invasiveness and heterogeneity limitations of tissue biopsies by analyzing tumor-derived molecules — such as ctDNA, cell-free DNA, circulating tumor cells, exosomes, and protein biomarkers — in blood or urine, thereby enabling non-invasive and dynamic monitoring (9). Among these, blood-based ctDNA has emerged as a research focus due to its ability to carry tumor-specific genetic information and ease of accessibility. ctDNA comprises fragmented DNA molecules released into body fluids during tumor cell apoptosis or necrosis, exhibiting high genetic concordance with primary tumor tissues (10). Compared with traditional protein biomarkers, ctDNA offers significant metabolic kinetic advantages, including a short half-life (approximately 2 hours), allowing real-time reflection of tumor-burden dynamics (11).

Although numerous clinical studies have confirmed the prognostic value of ctDNA in ICI-treated NSCLC patients, most are retrospective and lack high-level medical evidence. Therefore, this meta-analysis was conducted to synthesize and evaluate clinical evidence on the prognostic role of ctDNA in NSCLC patients receiving ICI therapy, providing a scientific foundation for clinical practice.

## 2 Method

The present study strictly adhered to the Preferred Reporting Items for Systematic Reviews and Meta-Analyses (PRISMA) guidelines (12) for reporting and assessed its methodological quality using guidelines of the Assessing the Methodological Quality of Systematic Reviews (13). Additionally, the study protocol was registered in the PROSPERO database (Registration ID: CRD420251025308).

### 2.1 Search strategy

Literature databases including PubMed, Embase, Cochrane Library, Web of Science, China National Knowledge Infrastructure (CNKI), and Wanfang Database were scanned (six databases in total). The search timeframe spanned the period from their inception to August 2025. Keywords such as “circulating tumor DNA”, “lung cancer”, and related terms were employed (details of the search strategy are provided in **Supplementary Table 1**). To maximize the comprehensiveness of retrieval, specific terms (e.g. “NSCLC” and “immunotherapy”) were deliberately excluded from the initial search strings. Subsequent manual screening was performed to identify studies focused on ICI therapy in NSCLC from all lung cancer studies involving ctDNA detection.

### 2.2 Exclusion and inclusion criteria

Studies were included if they met all of the following criteria: (1) Population: enrolled patients with advanced NSCLC; (2) Intervention: patients received either monotherapy with ICIs or ICI-based combination therapies, with a minimum treatment duration of one cycle; (3) ctDNA assessment: ctDNA analysis was performed before, during, or after ICI treatment; (4) Outcome reporting: available prognostic data included progression-free survival (PFS) and/or overall survival (OS); (5) Statistical metrics: reported hazard ratios (HRs) with corresponding 95% confidence intervals (95% CI) and P-values for PFS or OS; (6) Study selection: if multiple studies used overlapping cohorts, only the study with the largest sample size was included.

Studies were excluded based on any of the following: (1) Publication type: reviews, case reports, conference abstracts, Phase-I clinical trials, or non-English language publications; (2) Quality control: low-quality studies with Newcastle-Ottawa Scale or Cochrane RoB-2 scores below predetermined thresholds; (3) Data integrity: inability to extract valid outcome data or statistical metrics (e.g. missing HRs, 95% CI, or P-values); (4) Duplication: redundant publications or overlapping clinical trial data.

### 2.3 Data extraction

To ensure comprehensive and accurate retrieval of relevant studies, all initially identified records were subjected to independent screening by two researchers according to predefined inclusion and exclusion criteria, with any disagreements resolved through consultation with a third reviewer. Data extraction for included studies included key characteristics (first author, publication year, study design, and geographical region) alongside population demographics (sample size, median age, gender distribution, histologic subtypes, tumor stage, smoking history, PD-L1 expression, Eastern Cooperative Oncology Group performance status, treatment line, and therapeutic regimens), ctDNA-specific parameters (extraction methodology, timing of sampling, specimen type, sequencing approach, sequencing platform, and maximum variant allele frequency), and survival outcomes (HRs for PFS or OS with corresponding 95% CI and P-values), as well as trial-specific definitions of ctDNA response metrics.

### 2.4 Quality evaluation standards

For randomized controlled trials (RCTs), the Cochrane RoB-2 tool (14) was used to evaluate bias risk across six domains: random sequence generation, allocation concealment, blinding of participants and personnel, blinding of outcome assessment, incomplete outcome data, and selective outcome reporting, with each domain rated as “low risk”, “high risk”, or “unclear risk” of bias. For cohort studies, the NOS (15) assesses bias risk through three domains: selection (representativeness of cohorts and ascertainment of exposure), comparability (control for confounders), and outcome (assessment method and follow-up adequacy). Studies were scored on a 9-point scale, with total scores below 4, 4–6, and ≥ 7 indicating a high risk, moderate risk, and low risk of bias.

### 2.5 Statistical analysis methods

Review Manager 5.4 was used to calculate the HRs and their standard errors, while assessing between-study heterogeneity using Cochran’s Q-test and the I^2^ statistic; based on these results, an appropriate model was selected for pooled effect estimation — specifically, a random-effects model was chosen if P < 0.05 and I^2^ > 50%, whereas a fixed-effect model was applied if P > 0.05 and I^2^ ≤ 50% (16). To mitigate the influences of high-heterogeneity studies, inverse-variance weighting was implemented to assign weights to individual effect estimates. Forest plots were generated to visualize the pooled effect sizes, and funnel plots, together with Begg’s and Egger’s tests, were used to evaluate potential publication bias (17). Subsequently, StataMP 18 software was employed to perform sensitivity analysis leveraging the leave-one-out method (18).

## 3 Results

### 3.1 Study selection and quality assessment

**Figure 1:**
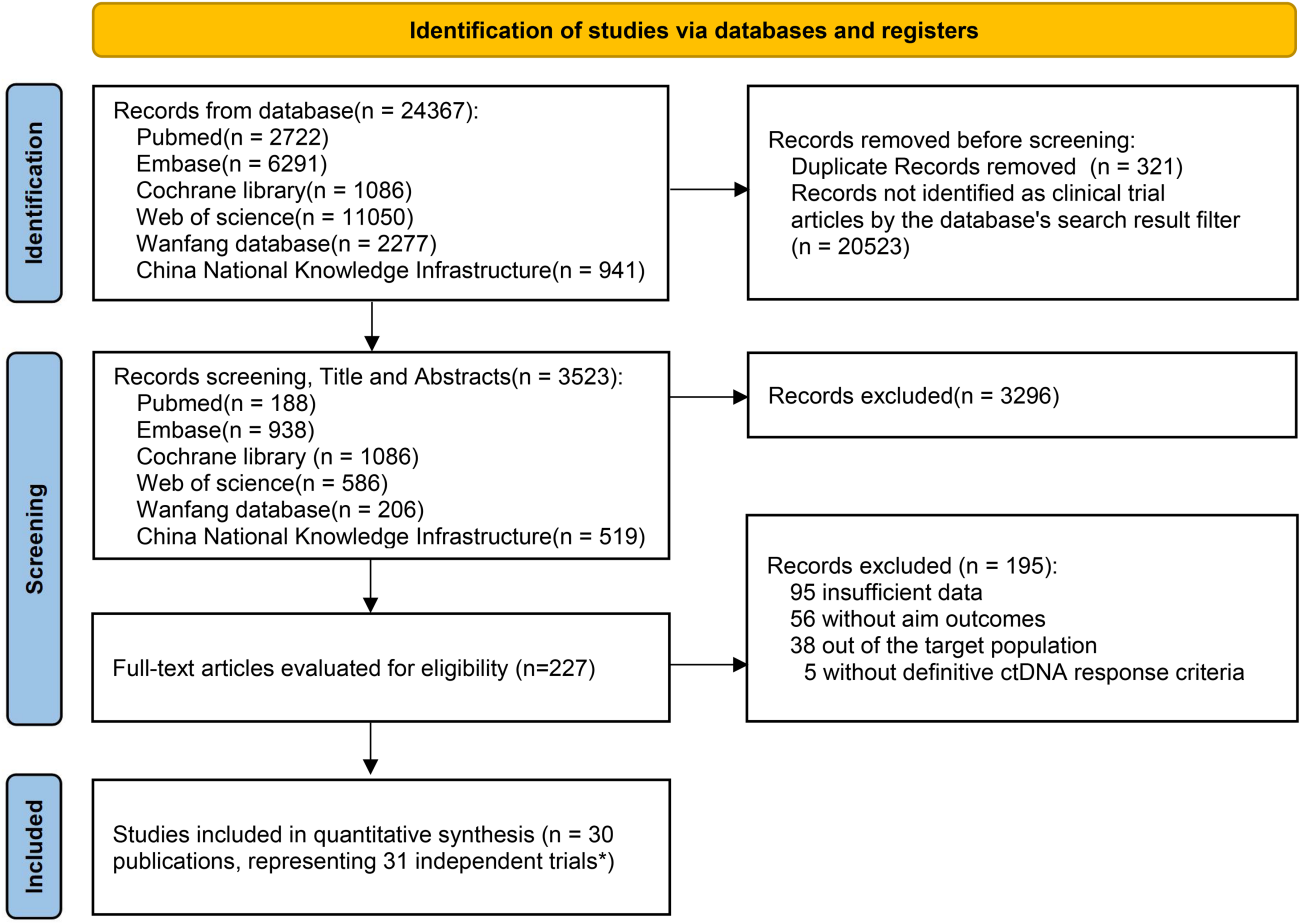
PRISMA flowchart used in study selection. *One publication (Raja R 2018) included two independent trials (ATLANTIC and study 1108), so the total number of trials included in the analysis is 31.

The initial systematic search yielded 24,367 potentially relevant articles, from which 321 duplicate records were removed; utilizing database-specific filters, 20,523 records not identified as clinical trial articles were excluded, leaving 3523 clinical trial records from PubMed (n = 188), Embase (n = 938), Cochrane Library (n = 1086), Web of Science (n = 586), Wanfang Database (n = 206), and CNKI (n = 519). Following title/abstract screening, 3,296 articles were excluded for failing to meet inclusion criteria. Full-text assessment of the remaining 227 articles led to the exclusion of 195 articles due to insufficient data (n = 95), lack of predefined endpoints (n = 56), population mismatch (n = 38), or undefined ctDNA response criteria (n = 5). After rigorous screening, 30 articles were included in this meta-analysis, with the study by Raja et al. (36) covering both the ATLANTIC and Study 1108 clinical trials. The Preferred Reporting Items for PRISMA flow diagram outline the study selection process (**Figure 1**).

The 31 included studies comprised 28 cohort studies and 3 RCTs. All cohort studies achieved NOS scores of 6–8 (rated as moderate-to-low risk), while the three RCTs also evaluated as low-risk according to Cochrane criteria. Further assessment details and scores are provided in **Supplementary Table 3**.

### 3.2 Study characteristics

The meta-analysis incorporated 31 clinical studies (**Table 1**) encompassing 2107 patients with stage advanced NSCLC treated with ICIs, with publication years spanning 2018 to 2025 (three studies in 2018, two in 2019, four in 2020, five in 2021, five in 2022, four in 2023, six in 2024, and two in 2025). Geographically, all studies were conducted in Europe or Asia (19 in Europe, 12 in Asia), utilizing plasma samples for ctDNA extraction and employing either next-generation sequencing (NGS) or droplet digital polymerase chain reaction (ddPCR) methodologies (28 utilizing NGS, three using ddPCR). Among the. 11 studies covered analysis of the association between baseline ctDNA status and PFS, while eight studies evaluated its relationship with OS; additionally, 25 studies investigated the correlation between ctDNA molecular dynamics and PFS, and 19 studies examined molecular response in relation to OS. Details of the characteristics of these clinical studies and associated data are presented in **Supplementary Table 1**.

**Table 1:** Characteristics of included clinical studies.

First author and year of publication	Region	Sample size	Line of therapy (number)	Detection method
Ai X 2024 (19)	China	83	1 (83), >1 (0)	NGS
Anagnostou V 2019 (20)	US	24	–	NGS
Anagnostou V 2023 (21)	US	50	1 (46), >1 (4)	NGS
Bruna Pellini 2023 (22)	US	117	1 (117), >1 (0)	NGS
Chen Y 2020 (23)	China	22	1 (0), >1 (22)	NGS
Chiang AC 2025 (24)	US	52	1 (49), >1 (3)	NGS
Goldberg SB 2018 (25)	US	28	1 (16), >1 (12)	NGS
Guibert N 2019 (26)	French	97	1 (0), >1 (97)	NGS
Han X 2022 (27)	China	33	1 (0), >1 (33)	NGS
Jia L 2025 (28)	China	20	1 (0), >1 (20)	NGS
Jia Q 2020 (29)	China	9	–	NGS
Jun S 2024 (30)	US	38	1 (38), >1 (0)	NGS
Kim ES 2022 (31)	US	152	1 (152), >1 (0)	NGS
Malene S 2022 (32)	Denmark	75	1 (75), >1 (0)	ddPCR
Murray JC 2024 (33)	US	30	1 (20), >1 (10)	NGS
Nabet BY 2020 (34)	US	99	1 (41), >1 (58)	NGS
Provencio M 2022 (35)	Spanish	46	1 (46), >1 (0)	NGS
Raja R 2018 (ATLANTIC) (36)	US	72	1 (0), >1 (72)	NGS
Raja R 2018 (Study 1108) (36)	US	28	1 (7), >1 (21)	NGS
Ricciuti B 2021 (37)	US	45	1 (45), >1 (0)	NGS
Su C 2021 (38)	China	92	1 (36), >1 (56)	NGS
Thompson JC 2021 (39)	US	67	–	NGS
van der Leest P 2021 (40)	Holland	100	1 (25), >1 (75)	ddPCR
Wang B 2022 (41)	China	9	1 (6), >1 (3)	NGS
Weber S 2021 (42)	Holland	167	1 (13), >1 (154)	NGS
Xu J 2024 (43)	China	57	1 (57), >1 (0)	NGS
Yang F 2023 (44)	China	44	1 (14), >1 (30)	NGS
Zhang M 2024 (45)	China	46	1 (46), >1 (0)	NGS
Zhong H 2023 (46)	China	62	1 (0), >1 (62)	NGS
Zhong J 2024 (47)	China	309	1 (309), >1 (0)	NGS
Zulato E 2020 (48)	Italy	34	–	ddPCR

### 3.3 The association between baseline ctDNA status and clinical outcomes

**Figure 2:**
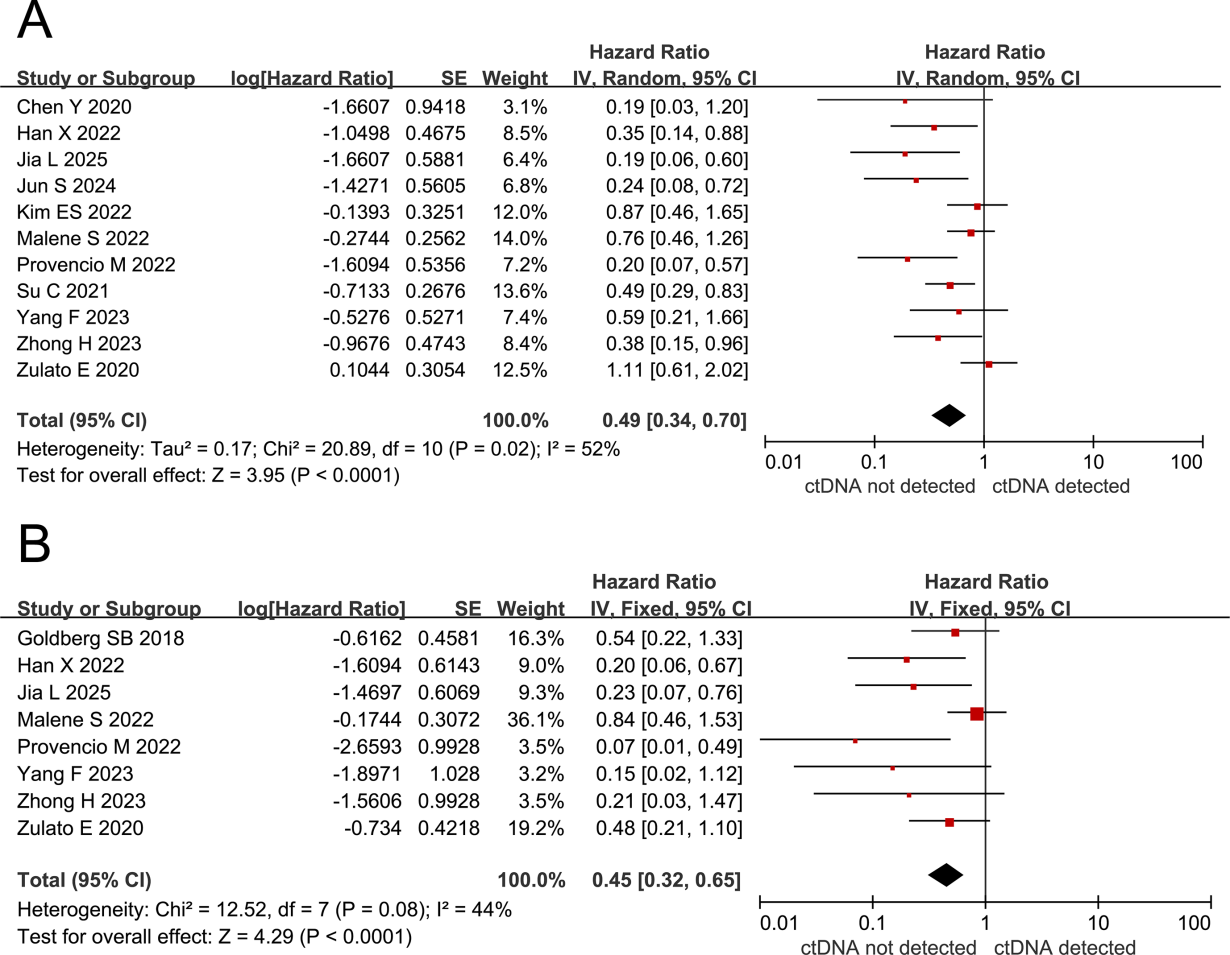
Forest plots for the association between baseline ctDNA status and clinical outcomes. With subfigures: **(A)** PFS, **(B)** OS.

For the PFS analysis incorporating 11 studies examining baseline ctDNA status before immunotherapy, significant between-study heterogeneity was observed (P = 0.02, I^2^ = 52%), prompting the use of a random-effects model. The pooled HR was 0.49 (95% CI: 0.34–0.70, P < 0.01), suggesting that the patients with undetectable ctDNA at baseline had significantly improved PFS compared to those with detectable ctDNA (**Figure 2A**).

Regarding OS, analyses from eight included studies demonstrated moderate heterogeneity (P = 0.08, I^2^ = 44%), leading to the application of a fixed-effects model. The pooled HR for OS was 0.45 (95% CI: 0.32–0.65, P < 0.01), further confirming statistically significant survival benefits in the patients without baseline ctDNA detection (**Figure 2B**).

Subgroup analyses (**Table 2**) confirmed consistent associations between baseline ctDNA status and clinical outcomes across geographic regions. Both Asian and European cohorts demonstrated significant correlations (all HRs P < 0.01), with no significant heterogeneity observed (P > 0.05 for interaction). Regarding treatment regimens, the patients receiving combination immunotherapy exhibited significantly improved survival benefits specifically when baseline ctDNA was undetectable (P < 0.01). No significant association was found in the monotherapy group (P > 0.05). Baseline ctDNA demonstrated enhanced predictive efficacy in later-line therapy patients (≥ 2nd line) compared to first-line cohorts, evinced by a lower HR to achieve statistical significance (P < 0.01). Methodologically, NGS showed significantly superior predictive sensitivity over ddPCR (interaction P < 0.01), whereas the ddPCR group failed to reach statistical significance (P > 0.05).

**Table 2:** Subgroup analyses of baseline ctDNA status for PFS and OS.

Subgroups	PFS				OS			
	HR(95% CI)	*P*	I^2^	Test for subgroup difference	HR(95% CI)	*P*	I^2^	Test for subgroup difference
Research area								
Asia	0.35 [0.21, 0.56]	<0.01	0%	P = 0.27	0.20 [0.10, 0.42]	<0.01	0%	P = 0.22
Europe	0.51 [0.31, 0.82]	<0.01	58%		0.36 [0.21, 0.62]	<0.01	44%	
Medication regimen								
immune monotherapy	0.99 [0.64, 1.35]	0.96	0%	P <0.01	0.48 [0.21, 1.10]	0.08	–	P = 0.09
combination therapy	0.27 [0.17, 0.44]	<0.01	0%		0.18 [0.09, 0.38]	<0.01	0%	
Treatment line								
first-line	0.49 [0.25, 0.95]	0.03	67%	P = 0.2	0.29 [0.03, 3.23]	0.31	83%	P = 0.97
Second-line and above	0.26 [0.13, 0.51]	<0.01	0%		0.28 [0.11, 0.70]	<0.01	64%	
Detection method								
NGS	0.40 [0.28, 0.58]	<0.01	30%	P <0.01	0.32 [0.20, 0.50]	<0.01	3%	P = 0.03
ddPCR	0.89 [0.60, 1.31]	0.55	55%		0.68 [0.40, 1.16]	0.16	13%	

### 3.4 The association between ctDNA molecular dynamics and clinical outcomes

**Figure 3:**
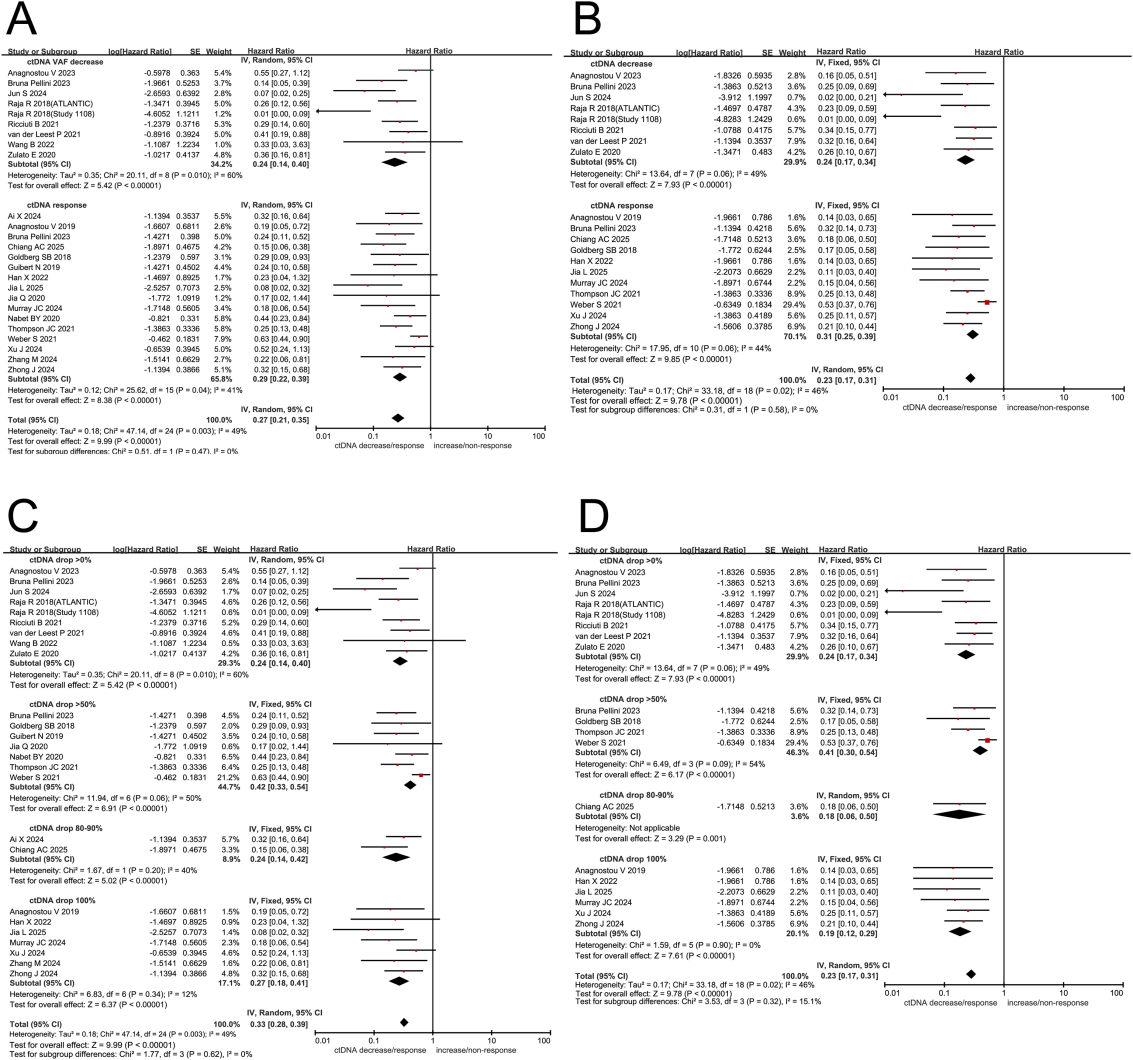
Forest plots for the association between ctDNA molecular kinetics and clinical outcomes. With subfigures: **(A)** PFS based on ctDNA reduction/response, **(B)** OS based on ctDNA reduction/response, **(C)** Stratified analysis for PFS by the level of VAF reduction, **(D)** Stratified analysis for OS by the level of VAF reduction.

Further exploration of ctDNA molecular dynamics revealed significant associations with clinical outcomes. Dynamic changes were classified into ctDNA reduction/increase and ctDNA response/no-response, wherein ctDNA reduction was defined as any decrease (variant allele frequency, VAF > 0%), while response definitions varied across studies — predominantly characterized as complete molecular clearance (100% VAF reduction) or > 50% VAF decrease, with minor variations including > 80% or > 90% reductions. Pooled analysis of 25 studies demonstrated that the patients achieving ctDNA reduction or response exhibited significantly improved PFS (HR = 0.27, 95% CI: 0.21–0.35; I^2^ = 49%, P < 0.01) (**Figure 3A**). Similarly, consolidated data from 19 studies confirmed markedly prolonged OS in these patients (HR = 0.23, 95% CI: 0.17–0.31; I^2^ = 46%, P < 0.01) (**Figure 3B**).

To address heterogeneity arising from divergent response thresholds, stratified analyses by the degree of VAF reduction were conducted: for PFS, HRs were 0.24 (> 0% reduction), 0.42 (> 50%), 0.24 (> 80%), and 0.27 (100% clearance) — all findings were statistically significant (P < 0.01), confirming consistent survival benefits regardless of the degree of reduction (**Figure 3C**). Equivalent OS analyses yielded HRs of 0.24 (> 0%), 0.41 (> 50%), 0.18 (> 80%), and 0.19 (100% clearance), with P < 0.01 throughout, establishing significant survival advantages across all molecular response thresholds (**Figure 3D**).

The subgroup analysis (**Table 3**) demonstrated the universal prognostic value of ctDNA molecular kinetics as a predictive biomarker across diverse clinical contexts in advanced cancer patients. The study revealed consistent associations between ctDNA dynamics and significant improvements in both PFS and OS, irrespective of patient ethnicity (Asian or European), treatment regimen (immune monotherapy or combination therapy), or line of therapy (first-line or previously treated stages). These correlations remained robust despite variations in the definition of ctDNA clearance thresholds, with all subgroup HR comparisons reaching statistical significance (P < 0.01). Further stratification based on VAF reduction thresholds implied that stricter criteria were associated with superior prognostic performance. The complete clearance (100% clearance) group was found to have the lowest heterogeneity (PFS I^2^ = 12%; OS I^2^ = 0%) and improved survival outcomes (PFS HR = 0.26; OS HR = 0.19).

**Table 3:** Subgroup analyses of ctDNA molecular kinetics for PFS and OS.

Subgroups	PFS				OS			
	HR(95% CI)	*P*	I^2^	Test for subgroup difference	HR(95% CI)	*P*	I^2^	Test for subgroup difference
Region								
Asia	0.30 [0.21, 0.44]	<0.01	0%	P = 0.59	0.20 [0.12, 0.32]	<0.01	0%	P = 0.55
Europe	0.27 [0.19, 0.37]	<0.01	61%		0.23 [0.17, 0.33]	<0.01	52%	
Medication regimen								
immune monotherapy	0.34 [0.21, 0.56]	<0.01	64%	P = 0.36	0.24 [0.13, 0.47]	<0.01	71%	P = 0.77
combination therapy	0.25 [0.16, 0.39]	<0.01	22%		0.22 [0.15, 0.31]	<0.01	0%	
Treatment line								
first-line	0.26 [0.19, 0.38]	<0.01	26%	P = 0.75	0.25 [0.17, 0.37]	<0.01	10%	P = 0.34
Second-line and above	0.24 [0.15, 0.39]	<0.01	2%		0.17 [0.09, 0.34]	<0.01	0%	
Detection method								
NGS	0.26 [0.20, 0.35]	<0.01	52%	P = 0.22	0.21 [0.15, 0.31]	<0.01	53%	P = 0.32
ddPCR	0.39 [0.22, 0.67]	0.55	0%		0.30 [0.17, 0.52]	0.55	0%	
Definition of ctDNA response								
VAF drop >0%	0.24 [0.14, 0.40]	<0.01	60%	P = 0.62	0.24 [0.17, 0.34]	<0.01	49%	P = 0.32
VAF drop >50%	0.42 [0.33, 0.54]	<0.01	50%		0.41 [0.30, 0.54]	<0.01	54%	
VAF drop 80-90%	0.24 [0.14, 0.42]	<0.01	40%		0.18 [0.06, 0.50]	<0.01	–	
VAF 100% clearance	0.27 [0.18, 0.41]	<0.01	12%		0.19 [0.12, 0.29]	<0.01	0%	

### 3.5 Publication bias evaluation

**Figure 4:**
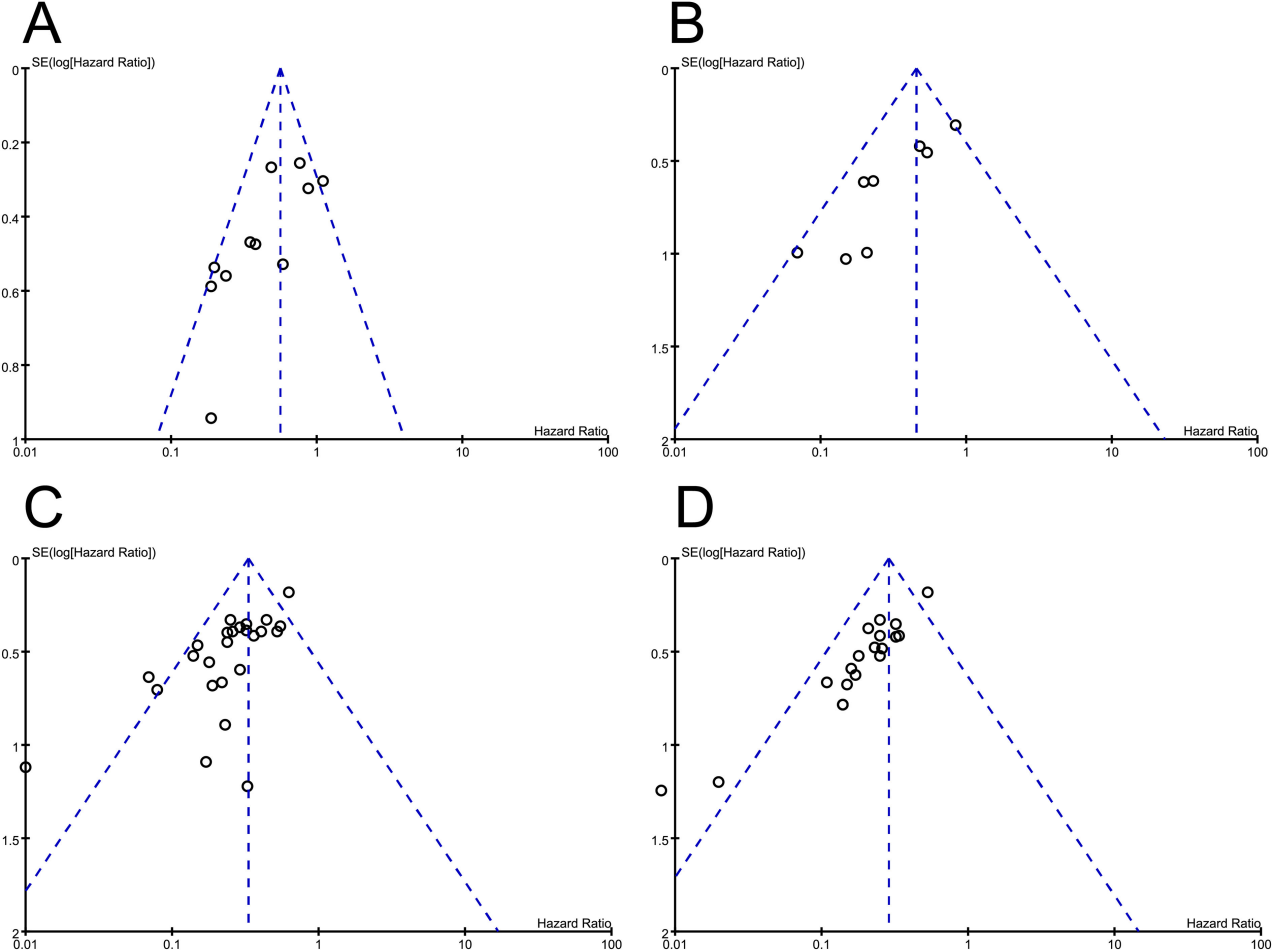
Publication bias evaluation using funnel plots. With subfigures: **(A)** Funnel plot for the association between baseline ctDNA status and PFS. **(B)** Funnel plot for the association between baseline ctDNA status and OS. **(C)** Funnel plot for the association between ctDNA molecular kinetics and PFS. **(D)** Funnel plot for the association between ctDNA molecular kinetics and OS.

To comprehensively evaluate publication bias, Deeks’ funnel plots (**Figure 4**) were constructed for the studies included in the four key meta-analyses described above, supplemented by quantitative Begg’s and Egger’s tests. The results revealed distinct patterns across analyses. Specifically, the funnel plot for the association between baseline ctDNA status and PFS (**Figure 4A**) demonstrated optimal characteristics, with data points distributed symmetrically along the central axis, forming a balanced funnel shape. This suggests a low risk of publication bias for this specific analysis.

In contrast, the funnel plots for baseline ctDNA status and OS (**Figure 4B**) and for ctDNA kinetics with PFS/OS (**Figures 4C, D**) exhibited mild to moderate left-skewness. These asymmetries suggest potential bias possibly arising from small-study effects or inter-study heterogeneity. Notably, all data points across the four plots remained within the funnel boundaries, with no extreme outliers detected, thereby ensuring the generalizability of the findings.

Quantitatively, Begg’s and Egger’s tests (**Table 4**) yielded statistically significant results for all four analyses (P< 0.05), confirming the presence of funnel plot asymmetry and raising concerns regarding potential publication bias. However, we refrained from applying the trim-and-fill method for correction. This decision was based on the algorithm’s failure to identify any missing studies suitable for “trimming” within the current dataset—a common occurrence in highly heterogeneous data. Consequently, we inferred that the observed asymmetry is more likely attributable to clinical or methodological heterogeneity across studies rather than pure publication bias.

**Table 4:** The results of Begg’s and Egger’s tests.

Meta-analysis	Begg’s Test *P*	Egger’s test *P*
The association between baseline ctDNA status and PFS	0.029	0.010
The association between baseline ctDNA status and OS	0.061	0.001
The association between ctDNA molecular kinetics and PFS	0.010	0.001
The association between ctDNA molecular kinetics and OS	0.001	0.001

Therefore, instead of relying on hypothetical ‘filled’ studies derived from the trim-and-fill method to adjust our results, we performed leave-one-out sensitivity analyses. This approach involved sequentially omitting individual studies to determine whether the overall conclusions were driven by specific studies or sources of heterogeneity.

### 3.6 Leave-one-out sensitivity analysis

**Figure 5:**
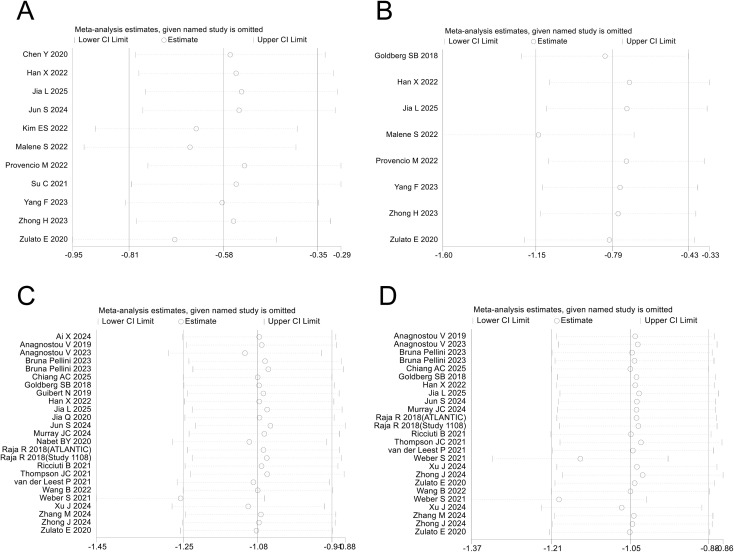
Sensitivity analysis of pooled HRs. With subfigures: **(A)** Leave-one-out sensitivity analysis of the association between baseline ctDNA status and PFS. **(B)** Leave-one-out sensitivity analysis of the association between baseline ctDNA status and OS. **(C)** Leave-one-out sensitivity analysis of the association between ctDNA molecular kinetics and PFS. **(D)** Leave-one-out sensitivity analysis of the association between ctDNA molecular kinetics and OS.

The robustness of four meta-analyses was evaluated through sensitivity analyses. As shown in the four subplots (**Figure 5**), the effect size estimates of all studies remained within relatively stable ranges, and overlapping confidence intervals indicated that individual studies exerted limited influence on the overall conclusions. Even if any single study was excluded, the direction of the pooled effect size was not reversed, and the 95% CI consistently remained within clinically meaningful ranges. These highly consistent sensitivity analysis results robustly demonstrate that conclusions of the meta-analyses in this study were not unduly affected by individual studies, thereby confirming their reliability.

## 4 Discussion

Existing meta-analyses on the clinical application of ctDNA in NSCLC largely focused on the predictive value in the perioperative setting (49–52) or during TKI therapy (53–56), with limited comprehensive evidence regarding its utility in immunotherapy (57, 58). Vega et al. (57) found that a tripartite VAF stratification (decrease/stable/increase) could predict outcomes in immunotherapy based on five clinical trials, and Wang et al. (58) synthesized 10 studies confirming early ctDNA reduction as a core indicator of treatment response and survival — effective across thresholds such as > 50% decline or any reduction — the present study substantially expands this evidence. The database search scope was broadened and recent studies not included in previous meta-analyses were incorporated, systematically integrating data from 31 global studies (2018–2025) involving 2,107 patients. This represents the largest and most up-to-date synthesis of the evidence (data current as of August 8, 2025) on the prognostic value of ctDNA in advanced NSCLC patients undergoing immunotherapy. Moreover, unlike earlier analyses, on-treatment ctDNA dynamics were refined using a four-tier VAF reduction stratification (> 0%, > 50%, > 80–90%, and 100% clearance). Subgroup analyses were also performed to validate consistency across regions (Asia/Europe), treatment regimens (monotherapy/combination), therapy lines (first-line/≥second-line), and detection technologies (NGS/ddPCR), providing robust evidence to support the translation of ctDNA from a biomarker into a clinical decision-making tool across diverse scenarios.

The meta-analysis demonstrates patients with undetectable baseline ctDNA had significant survival advantages, with HRs for PFS and OS of 0.49 and 0.45, respectively. Notably, the predictive power of baseline ctDNA levels for PFS was significantly stronger in the immunotherapy combination therapy group than the monotherapy (HR = 0.27 vs 0.99). This finding suggests that patients with detectable baseline ctDNA may benefit from more aggressive treatment strategies, such as immunotherapy combinations with anti-angiogenic agents or dual immunotherapy regimens. Furthermore, the predictive efficacy of baseline ctDNA levels and on-treatment molecular dynamics was more pronounced in the patients receiving second-line or later therapies, supporting the prioritization of dynamic ctDNA monitoring for optimizing later-line treatment decisions.

The dynamic changes in ctDNA during early treatment demonstrated stronger prognostic predictive power compared to a single baseline assessment. The HRs for molecular response of ctDNA (PFS: HR = 0.27; OS: HR = 0.23) were significantly more favorable compared to those for undetectable ctDNA at baseline (PFS: HR = 0.49; OS: HR = 0.45), highlighting the unique advantage of dynamic monitoring in capturing the biological effects of immunotherapy. This aligns with the recently proposed concept of “molecular response”, wherein early deep clearance of ctDNA serves as microscopic evidence of the successful immune system activation and effective tumor cell elimination (59). The advantage of such dynamic monitoring lies in its ability to detect “molecular progression” weeks or even months ahead of radiographic imaging (60), allowing for early intervention or treatment adjustment in patients at risk of progression, thus avoiding the toxicities and economic burdens associated with ineffective therapy. Furthermore, studies showed that in patients with radiographic progression (later confirmed as pseudo-progression), the ctDNA levels did not concurrently rise and often declined shortly after progression, indicating that dynamic ctDNA monitoring can distinguish true progression from pseudo-progression early in immunotherapy, thereby aiding clinical decisions on whether to continue treatment (61). In summary, dynamic ctDNA monitoring has become an indispensable component in optimizing clinical decision-making for immunotherapy.

At the technical level of ctDNA detection, it was found that NGS demonstrated significant prognostic value for predicting PFS and OS (P < 0.01), whereas ddPCR did not show statistical significance (P > 0.05). This discrepancy can be interpreted from two dimensions: on the one hand, only three ddPCR-related studies were included (accounting for 9.7% of the total), with a cumulative sample size of merely 209 cases, and insufficient statistical power may have caused a risk of false negatives; on the other hand, inherent limitations in the technical principles also contributed (compared to NGS) which can simultaneously track the clonal evolution of multiple genes; ddPCR targets only predefined single or limited mutations, resulting in systematic monitoring blind spots in the specific context of immunotherapy, which may also result in a large number of false negatives that dilute predictive efficacy (62, 63).

This systematic review and meta-analysis still requires careful consideration of several limitations. First, there was methodological heterogeneity among the included studies, including differences in detection technologies and platforms, as well as a lack of standardized sampling time points. The meta-analysis incorporated studies that utilized a variety of commercial or laboratory-developed testing protocols. These protocols differed in terms of the limit of detection (LOD), size of the targeted gene panel, sequencing depth, and the thresholds used to define “positive” or “detectable” ctDNA. A sample classified as “undetectable” on a platform with lower sensitivity may still harbor low-frequency ctDNA detectable on a more sensitive platform. This discrepancy in result interpretation due to varying LOD represents a significant source of clinical and technical heterogeneity in the present study, which could compromise the precision of the pooled effect size estimates. Second, variations in ctDNA response criteria, treatment regimens, and lines of therapy may have introduced additional bias into the results. Third, limited by the data structure in the published literature, raw data for some patients were not available, restricting in-depth analysis of confounding factors such as PD-L1 expression levels and the use of combined local therapy. Finally, Deeks’ funnel plot analysis suggested the presence of mild publication bias, primarily manifested as the clustering of small-sample studies (n < 30) on the left side of the effect size region. Finally, Deeks’ funnel plot analysis, together with Begg’s and Egger’s tests, suggested the presence of publication bias.

Despite these limitations, the present study improved the credibility of its conclusions through rigorous methodological design: a comprehensive search covered six major databases, and the use of both Cochrane RoB-2 and NOS quality evaluation systems ensured that all included studies met medium to high levels of evidence. It is noteworthy that sensitivity analysis verified that the direction of pooled HRs remained stable after excluding any individual study. Moreover, subgroup analyses based on detection technology, treatment regimen, geographic region, therapy line, and ctDNA response criteria revealed consistent patterns of predictive values of ctDNA across different scenarios: this multidimensional validation model provided strong support for the robustness of the results.

In summary, this study systematically validates the prognostic value of ctDNA in immunotherapy at the evidence-based medicine level, providing a scientific foundation for clinical decision-making. However, future research should prioritize the standardization of detection methods, including the establishment of unified sampling time points, sensitivity validation criteria, and standardized interpretation thresholds for negative/positive and molecular response, as well as encouraging the reporting of specific LOD parameters, to enable more reliable and comparable prognostic evaluations across different studies and technological platforms. Additionally, exploring epigenetic features of ctDNA — such as methylation profiles — may enhance the accuracy of prediction. Integrating ctDNA with other biomarkers (e.g. PD-L1 expression, and TMB) to build multidimensional predictive models, in tandem with rigorous validation through prospective clinical trials across diverse treatment settings (e.g. neoadjuvant therapy, first-line advanced disease, and resistance monitoring), will better guide personalized immunotherapy strategies.

The dynamic monitoring of ctDNA has shown potential for guiding treatment adjustments in clinical studies. For instance, according to the results of the APPLE trial, for patients with advanced EGFR-mutant NSCLC, dynamic monitoring of the T790M mutation in ctDNA during gefitinib treatment successfully identified molecular progression in 17% of patients prior to radiological progression. This earlier detection facilitated an earlier switch to osimertinib therapy, which consequently led to improved PFS and OS rates at 18 months (64). As detection technology advances and clinical evidence accumulates, the application of ctDNA in oncology is expanding. Moving forward, real-time monitoring of ctDNA is poised to enable more personalized therapeutic regimens, offering patients increasingly precise and dynamically optimized treatment pathways.

## 5 Conclusion

This meta-analysis definitively establishes that both baseline ctDNA levels and early-treatment molecular dynamics serve as reliable prognostic predictors of survival outcomes in advanced NSCLC patients receiving immunotherapy. Patients with undetectable baseline ctDNA or those achieving molecular response during therapy showed clinically substantial survival benefits, with this association being particularly evident in immune-combination therapy regimens. The study provides evidence-based support for incorporating ctDNA dynamic monitoring into clinical decision-making to optimize treatment strategies, underscoring its critical role in precision immunotherapy management.

## Data Availability

The original contributions presented in the study are included in the article/**Supplementary Material**. Further inquiries can be directed to the corresponding author.
