# Supplementary material for: Prognostic utility of circulating tumor DNA assessment in immune checkpoint inhibitor-treated advanced non-small cell lung cancer: a systematic review and meta-analysis
Source: Front Immunol. 2026 Jun 9;17:1812209. doi: 10.3389/fimmu.2026.1812209 (PMC13287079; doi:10.3389/fimmu.2026.1812209)
Supplement: Supplementary file 1 [file Table1.docx]

**Supplementary Table S1**

1. **Search strategy for Databases.**

| **Pubmed** | | |
| --- | --- | --- |
| **Number** | **Strategy** | **Results** |
| #1 | ("Lung Neoplasms"[Mesh]) OR ("Neoplasms, Pulmonary"[All Fields]) OR ("Neoplasm, Pulmonary"[All Fields]) OR ("Pulmonary Neoplasm"[All Fields]) OR ("Pulmonary Neoplasms"[All Fields]) OR ("Neoplasms, Lung"[All Fields]) OR ("Lung Neoplasm"[All Fields]) OR ("Lung Cancer"[All Fields]) OR ("Cancer, Lung"[All Fields]) OR ("Cancers, Lung"[All Fields]) OR ("Lung Cancers"[All Fields]) OR ("Cancer of Lung"[All Fields]) OR ("Pulmonary Cancer"[All Fields]) OR ("Cancer of Lung"[All Fields]) OR ("Pulmonary Cancer"[All Fields]) OR ("Cancer, Pulmonary"[All Fields]) OR ("Pulmonary Cancers"[All Fields]) OR ("Cancer of the Lung"[All Fields]) OR ("Cancers, Pulmonary"[All Fields]) OR ("Cancer of the Lung"[All Fields]) | 4,699 |
| #2 | ("Circulating Tumor DNA"[Mesh]) OR ("Residual Minimal Disease"[Mesh]) OR ("DNA, Circulating Tumor"[All Fields]) OR ("Tumor DNA, Circulating"[All Fields]) OR ("Cell-Free Tumor DNA"[All Fields]) OR ("Cell Free Tumor DNA"[All Fields]) OR ("DNA, Cell-Free Tumor"[All Fields]) OR ("Tumor DNA, Cell-Free"[All Fields]) OR ("Circulating Tumor DNA"[All Fields]) OR ("Minimal residual lesion"[All Fields]) OR ("MRD"[All Fields]) OR ("minimal residual disease"[All Fields]) OR ("Residual Neoplasms"[All Fields]) OR ("Minimal Disease, Residual"[All Fields]) OR ("Residual Minimal Disease"[All Fields]) OR ("Residual Minimal Diseases"[All Fields]) OR ("Residual Disease, Minimal"[All Fields]) OR ("Residual Neoplasms"[All Fields]) OR ("Minimal Residual Diseases"[All Fields]) OR ("Minimal Residual Disease"[All Fields]) OR ("Residual Neoplasm"[All Fields]) OR ("Residual Cancer"[All Fields]) OR ("Cancer, Residual"[All Fields]) OR ("Residual Cancer"[All Fields]) OR ("Residual Cancers"[All Fields]) OR ("Residual Tumor"[All Fields]) OR ("Residual Tumors"[All Fields]) OR ("Residual Tumour"[All Fields]) OR ("Residual Tumours"[All Fields]) OR ("Tumour, Residual"[All Fields]) | 385,078 |
| #3 | #1 AND #2 | 2,722 |
| **Embase** | | |
| **Number** | **Strategy** | **Results** |
| #1 | 'neoplasms, pulmonary':ab,ti OR 'neoplasm, pulmonary':ab,ti OR 'pulmonary neoplasm':ab,ti OR 'pulmonary neoplasms':ab,ti OR 'neoplasms, lung':ab,ti OR 'lung neoplasm':ab,ti OR 'lung cancer':ab,ti OR 'cancer, lung':ab,ti OR 'cancers, lung':ab,ti OR 'lung cancers':ab,ti OR 'cancer of lung':ab,ti OR 'pulmonary cancer':ab,ti OR 'cancer, pulmonary':ab,ti OR 'pulmonary cancers':ab,ti OR 'cancers, pulmonary':ab,ti OR 'cancer of the lung':ab,ti | 374,430 |
| #2 | ctdna:ab,ti OR 'circulating tumor':ab,ti OR 'tumor dna, circulating':ab,ti OR 'cell-free tumor dna':ab,ti OR 'cell free tumor dna':ab,ti OR 'dna, cell-free tumor':ab,ti OR 'tumor dna, cell-free':ab,ti OR 'circulating tumor dna':ab,ti OR 'minimal residual lesion':ab,ti OR mrd:ab,ti OR 'minimal disease, residual':ab,ti OR 'residual minimal disease':ab,ti OR 'residual minimal diseases':ab,ti OR 'residual disease, minimal':ab,ti OR 'residual neoplasms':ab,ti OR 'minimal residual diseases':ab,ti OR 'minimal residual disease':ab,ti OR 'residual neoplasm':ab,ti OR 'cancer, residual':ab,ti OR 'residual cancer':ab,ti OR 'residual cancers':ab,ti OR 'residual tumor':ab,ti OR 'residual tumors':ab,ti OR 'residual tumour':ab,ti OR 'residual tumours':ab,ti OR 'tumour, residual':ab,ti | 89,372 |
| #3 | #1 AND #2 | 6,291 |
| **Web of Science** | | |
| **Number** | **Strategy** | **Results** |
| #1 | TS=(Lung Neoplasms) OR AB=(Neoplasms, Pulmonary OR Neoplasm, Pulmonary OR Pulmonary Neoplasm OR Pulmonary Neoplasms OR Neoplasms, Lung OR Lung Neoplasm OR Lung Cancer OR Cancer, Lung OR Cancers, Lung OR Lung Cancers OR Cancer of Lung OR Pulmonary Cancer OR Cancer of Lung OR Pulmonary Cancer OR Cancer, Pulmonary OR Pulmonary Cancers OR Cancer of the Lung OR Cancers, Pulmonary OR Cancer of the Lung) | 716,819 |
| #2 | TS=(Circulating Tumor DNA OR Residual Minimal Disease) OR AB=(ctDNA OR DNA, Circulating Tumor OR Tumor DNA, Circulating OR Cell-Free Tumor DNA OR Cell Free Tumor DNA OR DNA, Cell-Free Tumor OR Tumor DNA, Cell-Free OR Circulating Tumor DNA OR Minimal residual lesion OR MRD OR minimal residual disease OR Residual Neoplasms OR Minimal Disease, Residual OR Residual Minimal Disease OR Residual Minimal Diseases OR Residual Disease, Minimal OR Residual Neoplasms OR Minimal Residual Diseases OR Minimal Residual Disease OR Residual Neoplasm OR Residual Cancer OR Cancer, Residual OR Residual Cancer OR Residual Cancers OR Residual Tumor OR Residual Tumors OR Residual Tumour OR Residual Tumours OR Tumour, Residual) | 113,185 |
| #3 | #1 AND #2 | 11,050 |
| **Cochrane Library** | | |
| **Number** | **Strategy** | **Results** |
| #1 | MeSH descriptor: [Circulating Tumor DNA] explode all trees | 108 |
| #2 | (Neoplasms, Pulmonary or Neoplasm, Pulmonary or Pulmonary Neoplasm or Pulmonary Neoplasms or Neoplasms, Lung or Lung Neoplasm or Lung Cancer or Cancer, Lung or Cancers, Lung or Lung Cancers or Cancer of Lung or Pulmonary Cancer or Cancer of Lung or Pulmonary Cancer or Cancer, Pulmonary or Pulmonary Cancers or Cancer of the Lung or Cancers, Pulmonary or Cancer of the Lung):ti,ab,kw (Word variations have been searched) | 4,042 |
| #3 | MeSH descriptor: [Lung Neoplasms] explode all trees | 36,754 |
| #4 | MeSH descriptor: [Medicine, Chinese Traditional] explode all trees | 11,179 |
| #5 | #2 or #3 | 37,094 |
| #6 | #1 or #4 | 11,179 |
| #7 | #5 and #6 | 1,086 |
| **China National Knowledge Infrastructure** | | |
| **Number** | **Strategy** | **Results** |
| #1 | ((SU=非小细胞肺瘤) OR (TKA=非小细胞肺癌+非小肺十肺癌+肺腺癌+肺鳞瘤+肺鳞状细胞癌+肺大细胞瘤+ LC十NSCLC(精确))) AND ((SU=循环肿瘤DNA) OR (TKA=循环肿瘤DNA +肿瘤循环DNA +循环肿瘤基因十肿瘤循环基因+ ctDNA十循环肿瘤细胞+ CTC + CTCS+游离DNA+循环DNA +血浆循环DNA +血浆游离DNA +血浆游离核酸+血液循环DNA(精确))) | 90,599 |
| #2 | ((SU=非小细胞肺瘤) OR (TKA=非小细胞肺癌+非小肺十肺癌+肺腺癌+肺鳞瘤+肺鳞状细胞癌+肺大细胞瘤+ LC十NSCLC(精确))) AND ((SU=循环肿瘤DNA) OR (TKA=循环肿瘤DNA +肿瘤循环DNA +循环肿瘤基因十肿瘤循环基因+ ctDNA十循环肿瘤细胞+ CTC + CTCS+游离DNA+循环DNA +血浆循环DNA +血浆游离DNA +血浆游离核酸+血液循环DNA(精确))) | 16,614 |
| #3 | #1 AND #2 | 941 |
| **Wanfang database** | | |
| **Number** | **Strategy** | **Results** |
| #1 | 主题:(循环肿瘤DNA) or 题名或关键词:(循环肿瘤DNA or 肿瘤循环DNA or 循环肿瘤基因 or 肿瘤循环基因 or ctDNA or 循环肿瘤细胞 or CTC or CTCs or 游离DNA or 循环DNA or 血浆循环DNA or 血浆游离DNA or 血浆游离核酸 or 血液循环DNA or 血液游离DNA or 血液游离核酸 or circulating tumor DNA or 无细胞状态的胞外DNA or 胞外DNA or 游离核酸分子 or 无细胞DNA or cfDNA or 液体活检 or liquid biopsy or 可测量残留病灶 or 分子残留病灶 or 微小残留病灶 or 肿瘤动力学)) | 35,698 |
| #2 | 主题:(非小细胞肺癌) or 题名或关键词:(非小细胞肺癌 or 非小肺 or 肺癌 or 肺腺癌 or 肺鳞癌 or 肺鳞状细胞癌 or 肺大细胞癌 or LC or NSCLC)) | 382,435 |
| #3 | #1 AND #2 | 2,277 |
